# Middle East respiratory syndrome coronavirus experimental transmission using a pig model

**DOI:** 10.1111/tbed.12668

**Published:** 2017-06-26

**Authors:** J. Vergara‐Alert, V. S. Raj, M. Muñoz, F. X. Abad, I. Cordón, B. L. Haagmans, A. Bensaid, J. Segalés

**Affiliations:** ^1^ IRTA Centre de Recerca en Sanitat Animal (CReSA, IRTA‐UAB) Bellaterra Spain; ^2^ Department of Viroscience Erasmus Medical Center Rotterdam The Netherlands; ^3^ UAB Centre de Recerca en Sanitat Animal (CReSA, IRTA‐UAB) Bellaterra Spain; ^4^ Facultat de Veterinària Departament de Sanitat i Anatomia Animals UAB Bellaterra, Barcelona Spain

**Keywords:** emerging diseases, MERS‐coronavirus (MERS‐CoV), Middle East respiratory syndrome (MERS), pig, transmission

## Abstract

Dromedary camels are the main reservoir of Middle East respiratory syndrome coronavirus (MERS‐CoV), but other livestock species (i.e., alpacas, llamas, and pigs) are also susceptible to infection with MERS‐CoV. Animal‐to‐animal transmission in alpacas was reported, but evidence for transmission in other species has not been proved. This study explored pig‐to‐pig MERS‐CoV transmission experimentally. Virus was present in nasal swabs of infected animals, and limited amounts of viral RNA, but no infectious virus were detected in the direct contact pigs. No virus was detected in the indirect contact group. Furthermore, direct and indirect contact pigs did not develop specific antibodies against MERS‐CoV. Therefore, the role of pigs as reservoir is probably negligible, although it deserves further confirmation.

## 1 INTRODUCTION

Middle East respiratory syndrome coronavirus (MERS‐CoV) was first detected in 2012 in Saudi Arabia, and it causes severe acute respiratory illness with fever, cough and shortness of breath (Zaki, van Boheemen, Bestebroer, Osterhaus, & Fouchier, 2012). Up to date, it has caused 1952 human infections, including 693 related deaths (World Health Organization (WHO), 2017). Dromedaries are the natural reservoir of MERS‐CoV (Sabir et al., 2016). However, other animal species such as non‐human primates (rhesus macaques and common marmosets), members of the family *Camelidae* (alpacas and llamas), rabbits and pigs have been demonstrated to be susceptible to MERS‐CoV infection (Crameri et al., 2016; Falzarano et al., 2014; Haagmans et al., 2015; Munster, de Wit, & Feldmann, 2013; Vergara‐Alert, van den Brand, et al., 2017; de Wit et al., 2013, 2017). The finding that pigs can be infected with MERS‐CoV would suggest that other *Suidae* might be susceptible to the virus. Indeed, common warthogs (*Phacochoerus africanus*), bushpig (*Potamochoerus larvatus*) and wild boars are commonly found in the Greater Horn of Africa or the Middle East, sharing the same habitats and water sources with dromedaries (Cumming, 2008; Vergara‐Alert, Vidal, Bensaid, & Segalés, 2017). A recent study in alpacas demonstrated efficient animal‐to‐animal transmission (Adney, Bielefeldt‐Ohmann, Hartwig, & Bowen, 2016) but, to our knowledge, evidence for transmission between animals from other species has not been reported. To study whether MERS‐CoV might be transmitted between pigs, an experimental transmission study in this animal model was designed and performed under direct and indirect contact settings.

## 2 MATERIALS AND METHODS

### 2.1 Experimental design

**Figure 1:**
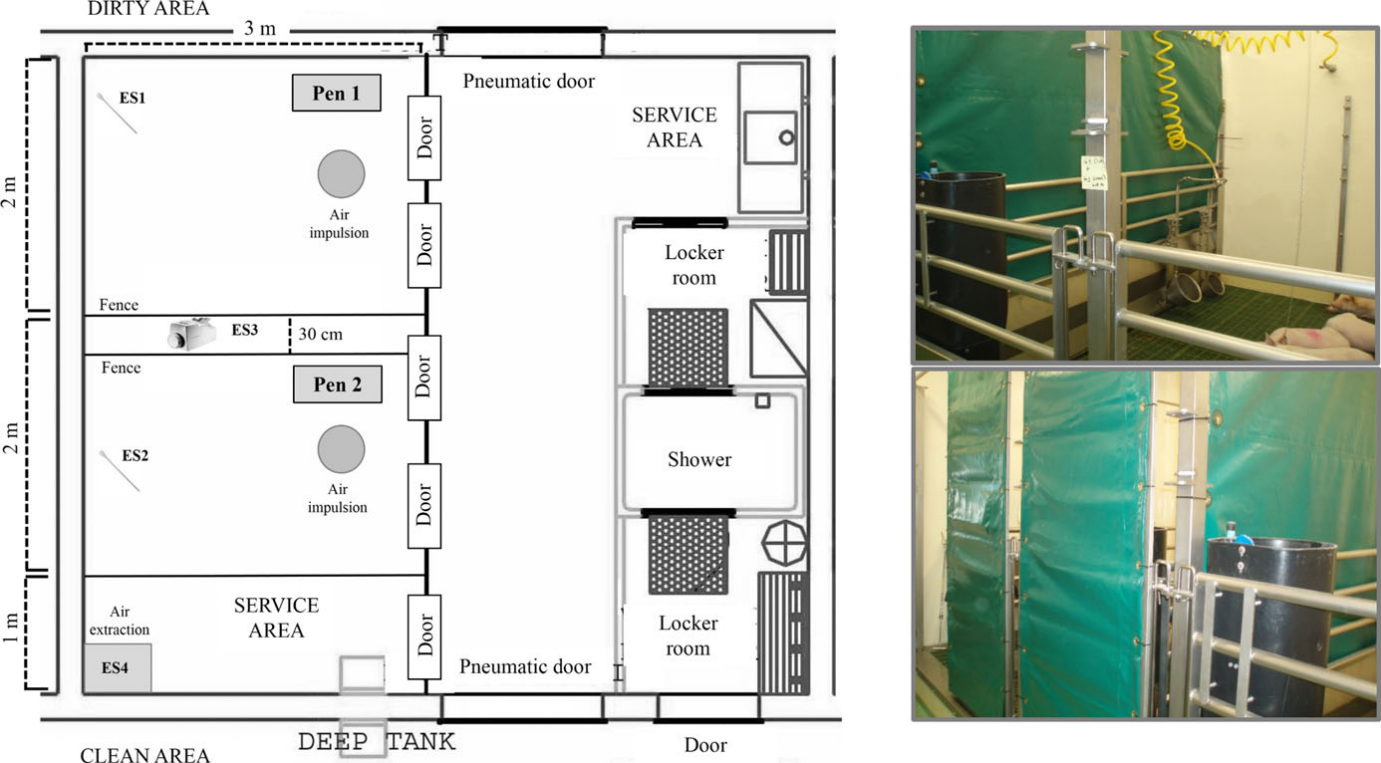
Schematic representation of the experimental animal box. Boxes in the animal facility of the biosafety level 3 at IRTA‐CReSA are behind two sets of doors (with a shower in between) following the standards of a negative pressure room. Animals were distributed into two pens separated by two fences with a 30 cm distance between them. G1 (P1–P5) was allocated in pen 1 and G2 (P6–P10) and G3 (P11–P15), in pen 2. Two days after inoculation of G1 with MERS‐CoV, G2 was cohoused with G1 until the end of the experiment. Tarpaulin was used to prevent contact between G1 and the other two groups during the firsts 2 days after inoculation. Environmental samples (ES) were obtained from different locations, as represented in the scheme

Fifteen six to eight‐week‐old Yorkshire × Landrace pigs (Ca N'Arola S.L., Castellterçol, Barcelona, Spain) were housed at Biosafety Level 3 (BSL‐3) animal facilities (IRTA‐CReSA, Barcelona, Spain), and divided into three groups: G1, MERS‐CoV‐inoculated pigs (P1–P5); G2, direct contacts (P6–P10); G3, indirect contacts (P11–P15). Three extra animals were used as negative controls (G4, P16–P18). Animals from G1, G2 and G3 were housed in the same experimental box unit but placed in two different pens. The pens were separated by two fences with a 30 cm distance among them (Figure 1). Tarpaulin, from the ceiling to the floor, was used to avoid contact between pen 1 and pen 2. Tarpaulin was also placed in the front doors of both pens. At the beginning of the experiment, G1 was housed in pen 1, and G2 and G3 in pen 2.

G1 was inoculated with 10^7^ TCID_50_ (50% tissue culture infectious dose) MERS‐CoV (passage 7 human isolate HCoV‐EMC/2012) in 3 mL saline solution via intranasal route (1.5 mL in each nostril). Two days later, all tarpaulins were removed and G2 pigs were moved from pen 2 to pen 1 until the end of the study.

All animals were monitored daily for clinical signs (sneezing, coughing, nasal discharge and/or dyspnoea), as well as rectal temperatures until day 10 post‐inoculation (PI). Nasal swabs (NS) were obtained on days 0, 1, 2, 3, 4 PI from all animals and at days 7, and 10 PI from G1. Animals from G2 and G3 were also sampled at 5, 6, 9 and 12 days PI, corresponding to days 3, 4, 7 and 10 after direct (G2) and indirect (G3) contact with G1.

### 2.2 Sampling procedures

Two independent NS were collected and placed in PBS (for PCR analysis) and DMEM containing antimicrobial drugs (for detection of infectious virus); swabbing was performed deep in both sides of the nasal cavity. Sera were obtained before challenge and at 7, 15 and 26 days PI, and they were subsequently used to detect the presence of MERS‐CoV‐specific antibodies. Negative control pigs were sampled (NS and sera) and euthanized before the start of the experiment.

Daily environmental samples (ES) between day 0 and 10 PI were obtained from air sampling and wall surface swabbing (Figure 1). Briefly, swabs pre‐moistened with transport medium (Copan Universal Transport Medium UTM‐RT System) were collected from walls in pen 1 and 2 (ES1 and ES2). Air sampling was performed using an air sampler (Airport MD8 Sartorius device) located between pens, which suctioned 50 L/min air volume for 20 min through a gelatin membrane filter (ES3). Air from the box unit was sampled with 10 × 10 cm dry membrane filters located in the air extraction of the box (ES4). ES were tested for the presence of viral RNA.

### 2.3 Virus detection

Viral RNA from NS and ES was extracted with NucleoSpin^®^ RNA virus kit (Macherey‐Nagel, Germany) following the manufacturer's instructions. The RNA extracts were tested by the UpE PCR (Raj et al., 2013), and the techniques were carried on as previously (Vergara‐Alert, van den Brand, et al., 2017). NS were also evaluated for the presence of infectious virus by titration in Vero cells, following previous protocol (Vergara‐Alert, van den Brand, et al., 2017).

### 2.4 Humoral immune response assays

Serum samples from days 0, 7, 15 and 26 PI were tested to determine the specific S1‐antibodies by a MERS‐CoV S1‐ELISA, and by a specific virus neutralization assay, as previously described (Haagmans et al., 2016).

## 3 RESULTS AND DISCUSSION

**Figure 2:**
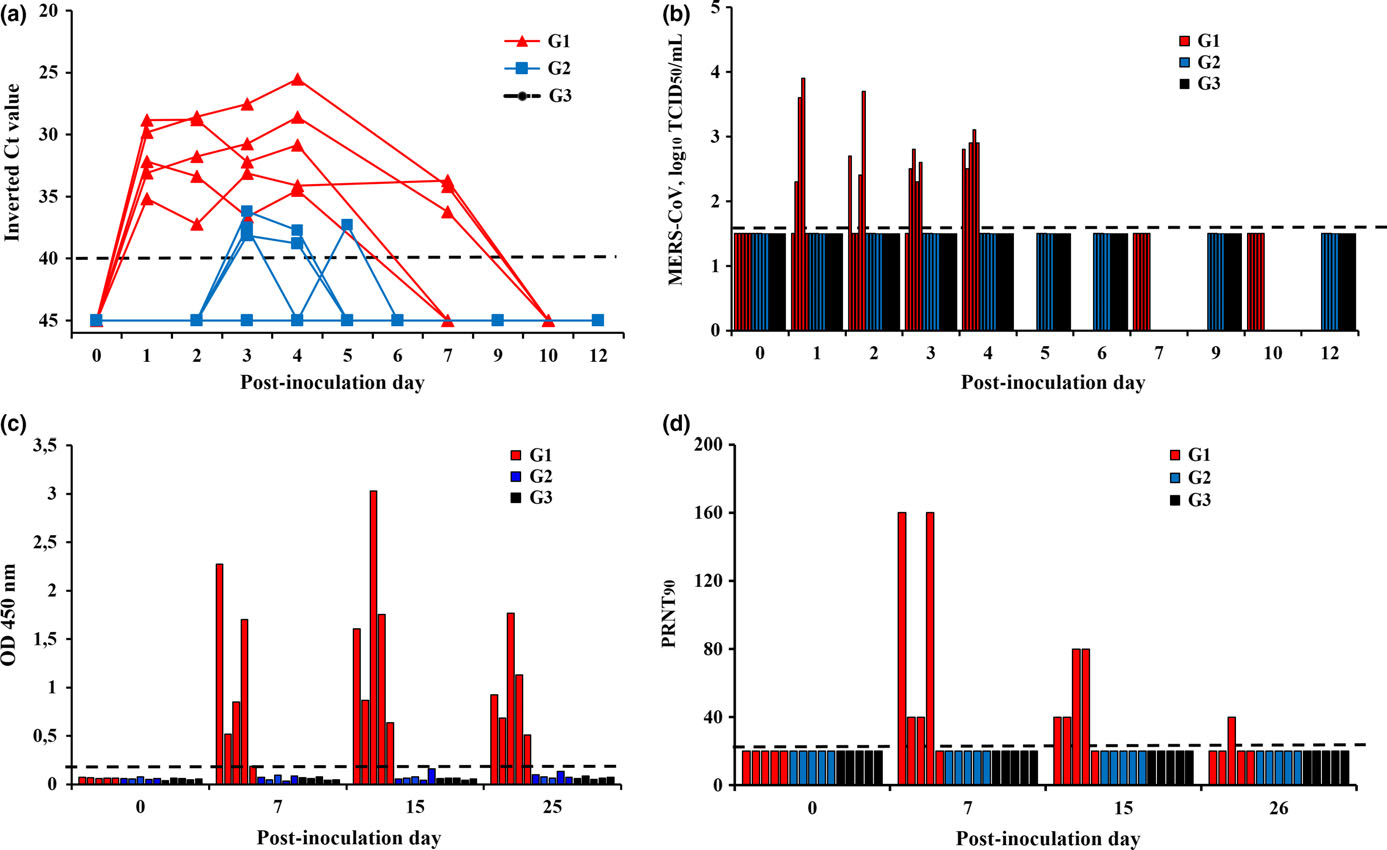
Viral shedding and antibody responses after experimental inoculation of MERS‐CoV into pigs (G1), and after direct (G2) or indirect (G3) exposure of non‐infected pigs with G1. (a) Viral RNA and (b) infectious MERS‐CoV from pigs nasal swab samples collected at different times after challenge. Each line or bar represents an individual animal. (c) MERS‐CoV S1 antibody responses were analysed in serum from all animals at post‐inoculation days 0, 7, 15 and 26. An ELISA with recombinant MERS‐CoV S1 protein was used, and results are represented individually. (d) Individual MERS‐CoV neutralization titres from pigs as determined from serum. Dashed lines depict the detection limit of the assays. Ct, cycle threshold; MERS‐CoV, Middle East respiratory syndrome coronavirus; OD, optical density; PRNT_90_, 90% plaque reduction neutralization test; TCID_50_, 50% tissue culture infective dose

Similar to a previous experiment (Vergara‐Alert, van den Brand, et al., 2017), none of the pigs had appreciable rise in rectal temperature upon challenge, nor any clinical signs (data not shown). All MERS‐CoV‐experimentally infected animals (P1–P5) shed viral RNA at least from 1 to 4 days PI, and three of five pigs had detectable viral RNA until 7 days PI (Figure 2a). Most importantly, all five animals shed infectious virus during the first 4 days PI (Figure 2b). Viral RNA was detected in four of five cohoused, direct contact animals (G2) at least one time PI. The MERS‐CoV RNA load of G2 pigs, however, was lower than those of G1 (Figure 2a). No viral RNA or infectious virus was detected in swabs from G3 (P11–P15) and control pigs (P16–P18).To test whether seroconversion occurred, serum samples were tested with a specific recombinant MERS‐CoV S1‐ELISA and for neutralizing antibodies against MERS‐CoV. All five MERS‐CoV infected animals (P1–P5) had detectable levels of S1‐antibodies 2‐ and 3‐weeks after the infection (Figure 2c). The specificity of the response was confirmed by virus neutralization assay. In P1–P4 (but not in P5), serum neutralizing MERS‐CoV‐specific titres (1:40–1:160) were detected at 1‐ and 2‐week PI (Figure 2d). However, at week 3 PI, the virus neutralizing antibodies decreased (1:20–1:40). No MERS‐CoV‐specific antibodies were detected in serum of G2, G3 and control pigs. In environmental samples, very low levels of viral RNA were detected at different time points, with a peak at day 5 PI (Table 1).

**Table 1:** Viral RNA from air sampling and wall surface swabbing at different times after MERS‐CoV infection. Swabs were collected from the walls in pen 1 and pen 2 (ES1 and ES2), air sampling was performed with an air device (ES3), and circulating air from the box was sampled with filters located in the ceiling air extraction of the room (ES4)

Sample	Post‐inoculation day										
	0	1	2	3	4	5	6	7	8	9	10
ES1	n.d.	40.26	40.86	n.d.	n.d.	36.35	n.d.	n.d.	n.d.	n.d.	n.d.
ES2	n.d.	n.d.	n.d.	n.d.	n.d.	n.d.	n.d.	n.d.	n.d.	n.d.	n.d.
ES3	n.d.	n.d.	n.d.	n.d.	n.d.	40.56	40.52	39.78	n.d.	n.d.	n.d.
ES4	n.d.	40.28	40.69	n.d.	n.d.	40.31	n.d.	39.87	n.d.	n.d.	n.d.

ES, environmental sample; n.d., non‐detected.

Other livestock besides dromedaries are susceptible to MERS‐CoV infection (Crameri et al., 2016; Falzarano et al., 2014; Haagmans et al., 2015; Munster et al., 2013; Vergara‐Alert, van den Brand, et al., 2017; de Wit et al., 2013, 2017); thus, they might be potential intermediate hosts of the virus. However, transmission studies have only been performed in alpacas. Here, no MERS‐CoV effective transmission was observed between pigs, as sustained by two main facts. First, viral RNA was detected from four of the five cohoused animals (G2), but at levels relatively similar to those found in environmental samples, and during a shorter period time than G1. Second, seroconversion was only observed in MERS‐CoV‐infected group pigs (G1), but specific antibodies were not detected in G2 or G3.

In summary, using our pig model of MERS‐CoV infection (Vergara‐Alert, van den Brand, et al., 2017), we analysed animal‐to‐animal transmission of MERS‐CoV. Although the role of *Suidae* in the transmission of MERS‐CoV should be further clarified, unlike camelids, pigs do not seem to be able to transmit MERS‐CoV efficiently, suggesting that the role of pigs as reservoir is probably negligible.
